# LincRNA-Gm4419 knockdown ameliorates NF-*κ*B/NLRP3 inflammasome-mediated inflammation in diabetic nephropathy

**DOI:** 10.1038/cddis.2016.451

**Published:** 2017-02-02

**Authors:** Hong Yi, Rui Peng, Lu-yu Zhang, Yan Sun, Hui-min Peng, Han-deng Liu, Li-juan Yu, Ai-ling Li, Ya-juan Zhang, Wen-hao Jiang, Zheng Zhang

**Affiliations:** 1 Department of Cell Biology and Medical Genetics, Chongqing Medical University, Chongqing, China; 2 Molecular Medicine and Cancer Research Center, Chongqing Medical University, Chongqing, China; 3 Department of Bioinformatics, Chongqing Medical University, Chongqing, China; 4 Experimental Teaching Center, Chongqing Medical University, Chongqing, China

## Abstract

Diabetic nephropathy (DN) as the primary cause of end-stage kidney disease is a common complication of diabetes. Recent researches have shown the activation of nuclear factor kappa light-chain enhancer of activated B cells (NF-*κ*B) and NACHT, LRR and PYD domain-containing protein 3 (NLRP3) inflammasome are associated with inflammation in the progression of DN, but the exact mechanism is unclear. Long noncoding RNAs (lncRNAs) have roles in the development of many diseases including DN. However, the relationship between lncRNAs and inflammation in DN remains largely unknown. Our previous study has revealed that 14 lncRNAs are abnormally expressed in DN by RNA sequencing and real-time quantitative PCR (qRT-PCR) in the renal tissues of db/db DN mice. In this study, these lncRNAs were verified their expressions by qRT-PCR in mesangial cells (MCs) cultured under high- and low-glucose conditions. Twelve lncRNAs displayed the same expressional tendencies in both renal tissues and MCs. In particular, long intergenic noncoding RNA (lincRNA)-Gm4419 was the only one associating with NF-*κ*B among these 12 lncRNAs by bioinformatics methods. Moreover, Gm4419 knockdown could obviously inhibit the expressions of pro-inflammatory cytokines and renal fibrosis biomarkers, and reduce cell proliferation in MCs under high-glucose condition, whereas overexpression of Gm4419 could increase the inflammation, fibrosis and cell proliferation in MCs under low-glucose condition. Interestingly, our results showed that Gm4419 could activate the NF-*κ*B pathway by directly interacting with p50, the subunit of NF-*κ*B. In addition, we found that p50 could interact with NLRP3 inflammasome in MCs. In conclusion, our findings suggest lincRNA-Gm4419 may participate in the inflammation, fibrosis and proliferation in MCs under high-glucose condition through NF-*κ*B/NLRP3 inflammasome signaling pathway, and may provide new insights into the regulation of Gm4419 during the progression of DN.

Diabetic nephropathy (DN) as the primary cause of end-stage kidney disease is a common complication of diabetes mellitus.^1^ In recent years, studies have indicated that the inflammatory pathways have central roles in the developmental and progressive stages of DN.^2, 3^ Inflammation is involved in the pathogenesis of DN through various pro-inflammatory cytokines, including monocyte chemoattractant protein-1 (mcp-1), tumor necrosis factor-*α* (TNF-*α*) and interleukin-1*β* (IL-1*β*) *in vivo* and *in vitro*.^4, 5, [Bibr bib6]^Moreover, transcription factor nuclear factor kappa light-chain enhancer of activated B cells (NF-*κ*B) is an important inflammatory stimulus for DN.^7^ NF-*κ*B inhibitor alpha (I*κ*B*α*) regulates activation of NF-*κ*B p50/p65 heterodimer,^8^ and p50/p65 subunits translocate into the nucleus and bind to specific promoter sequence of the target inflammatory genes when I*κ*B*α* is degraded by immunoproteasome.^9, 10^ Also, our previously study has shown that the activation of NF-*κ*B has a role in renal inflammation and fibrosis of the progression of DN.^3^ In addition, recent researches have displayed that NACHT, LRR and PYD domain-containing protein 3 (NLRP3) inflammasome directly participated in renal inflammatory processes, leading to the progression of diabetic glomerular damage, including glomerular fibrosis, hyperplasia of the extracellular matrix and glomerulosclerosis.^11, 12, 13, 14^ Studies also have shown that NF-*κ*B and NLRP3 inflammasome participated the inflammatory phase of many diseases including DN.^15, 16^ Therefore, these suggest that NF-*κ*B/NLRP3 inflammasome signaling pathway may have an important role in DN. However, the mechanism still remains largely mysterious in the progression of DN.

The eukaryotic genomes consists of not only plenty of protein-coding RNAs but also lots of noncoding RNAs (ncRNAs).^17, 18, 19^ The long noncoding RNA (lncRNA), defined simply on the basis of transcripts >200 nt and lack of protein-coding capability, is a new class of ncRNAs and has been found to be pervasively transcription in the genome, which mainly locate within the nucleus or cytosolic compartment.^20^ lncRNAs can be further classified into the following locus biotypes according to their locations with respect to protein-coding genes: intronic transcript lncRNAs, promoter-associated lncRNAs, antisense lncRNAs, long intergenic noncoding RNAs (lincRNAs) and UTR-associated lncRNAs.^21^ Although the vast majority of lncRNA functions are unknown, lncRNAs have emerged as a major category of regulatory eukaryotic transcripts.^22, 23^ Recently, lncRNAs have emerged as novel gene regulators and prognostic markers in several diseases including DN. However, the functions of lncRNAs in inflammatory processes in DN are still unclear. More importantly, NF-*κ*B is a critical link between inflammation and DN, but whether lncRNAs regulate its activation in DN remains unknown.

Recent studies utilizing high-throughput RNA sequencing (RNA-seq) technology and chromatin immunoprecipitation (ChIP) sequencing technology have identified thousands of abnormally expressed lncRNAs.^24^ By using RNA-Seq and real-time quantitative PCR (qRT-PCR), we recently identified 14 dysexpession lncRNAs in the kidney tissues of db/db DN mice including lncRNA-Gm4419. Thus, it indicates that Gm4419 may be involved in DN. However, up to now there is no report to show the association between Gm4419 and any diseases, including DN. In the present study, we further verify the expressions of 14 DN-related lncRNAs in mesangial cells (MCs) cultured under high-glucose condition by qRT-PCR. Results show that 12 lncRNAs express the same tendencies both in renal tissues and MCs. Among them, lincRNA-Gm4419 is the only one associating with NF-*κ*B by bioinformatics methods. Moreover, our study further suggests that Gm4419 knockdown could obviously ameliorate inflammation, fibrosis and proliferation in MCs with high glucose, but overexpression of Gm4419 could enhance inflammation, fibrosis and proliferation in MCs with low glucose. In addition, Gm4419 could activate NF-*κ*B pathway by directly interacting with p50, the subunit of NF-*κ*B, and p50 could directly interact with NLRP3 inflammasome in MCs. Therefore, our data display that Gm4419 may have important roles in inflammation, fibrosis and cell proliferation in DN via NF-*κ*B/NLRP3 inflammasome signaling pathway.

## Results

### Gm4419 is upexpressed in MCs cultured with high glucose and mainly distributes in the cytoplasm of MCs

**Figure 1:**
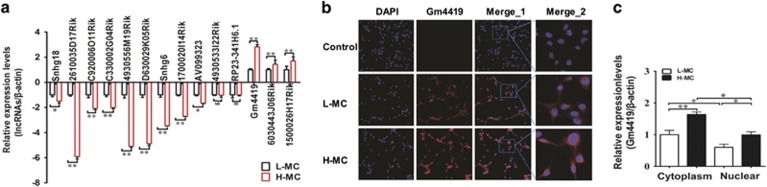
The expression and distribution of Gm4419 in MCs. (**a**) qRT-PCR analyzed 14 lncRNAs levels in MCs that expressional tendencies corresponded with RNA-seq in renal tissues of db/db DN mice, in response to high or low glucose (*n*=3). (**b**) Localization of Gm4419 (red) by FISH in MCs that were stimulated by high or low glucose. Gm4419 was mainly distributed in the cytoplasm of cells. Moreover, Gm4419 was weakly expressed in L-MCs, whereas it was highly expressed in H-MCs. Control group represented that MCs does not exist self-illumination (× 200 and × 800 in enlarged images). (**c**) The mRNA levels of Gm4419 were measured in the cytoplasm and nuclear of MCs by qRT-PCR. The data were representative of the results of three independent experiments, and the data were presented as means±S.E.M. (*P<0.05, **P<0.01, NS, no significant)

Our previous study showed that 14 lncRNAs were abnormally expressed in renal tissues of db/db DN mice by RNA-seq and qRT-PCR (data not shown). On the basis of these data, we verified the expressions of these 14 lncRNAs in MCs cultured under high- or low-glucose condition by qRT-PCR. Results revealed that two lncRNAs were not abnormally expressed, whereas 12 lncRNAs were abnormally expressed among these 14 lncRNAs in MCs, including 9 downexpressed and 3 upexpressed lncRNAs (Figure 1a). Moreover, we compared the differences of RNA-seq and qRT-PCR in renal tissues with the differences of qRT-PCR in MCs, data were showed in Table 1.

Then, Gm4419 was focused on for further study because it was not only the top one upexpressed lncRNA in DN but also the only lncRNA that had the potential binding sites with DN inflammation-related gene NF-*κ*B among these 12 lncRNAs by bioinformatics methods (Supplementary Table 1). In view of our results showed that Gm4419 was upexpressed in H-MCs, we further detected the subcellular location of Gm4419 in L-MCs and H-MCs. Fluorescence *in situ* hybridization (FISH) and qRT-PCR results (Figures 1b and c) showed that Gm4419 was expressed both in the nucleus and cytoplasm of L-MCs and H-MCs, but the expression of Gm4419 was markedly high in the cytoplasm when compared with that in the nucleus (*P*<0.05). It suggested that Gm4419 was predominantly distributed in the cytoplasm of MCs. Furthermore, our study displayed that Gm4419 expression was significantly higher in the cytoplasm of H-MCs than in the cytoplasm of L-MCs (*P*<0.05). Therefore, all above results revealed that Gm4419 may have a role in the cytoplasm of MCs.

### Gm4419 regulates inflammation, fibrosis and cell proliferation of MCs under high-glucose condition

To study the functional role and potential mechanisms of Gm4419 in DN, we first amplified the full length of Gm4419 using the rapid amplification of complementary DNA (cDNA) ends (RACE) and cloned the full-length sequence of Gm4419 into a pcDNA3.1 vector to construct a stable Gm4419 overexpression plasmid [Gm4419 (+)]. The plasmid was confirmed by gel electrophoresis and sequencing after restriction enzyme digestion (Supplementary Figure 1a). Moreover, the plasmid Gm4419 (+) or the empty plasmid pcDNA3.1 was transfected into L-MCs in which Gm4419 was poorly expressed. In addition, qRT-PCR and FISH results showed that Gm4419 (+) could largely increase Gm4419 expression when compared with that in L-MC mock and L-MC pcDNA3.1 groups (Supplementary Figures 1b and c). Meanwhile, Gm4419 knockdown was carried out in H-MCs in which Gm4419 was highly expressed by transfecting with Gm4419 Small interfering RNAs (siRNAs). As shown in Supplementary Figure 1b, the knockdown effect of siGm4419 (No. 3) was best compared with siGm4419 (No.1 and 2) by qRT-PCR. Thus, we used siGm4419 (No. 3) for further experiments. Furthermore, FISH data showed that siGm4419 was markedly downregulated when compared with that in H-MC mock and H-MC siNC groups (Supplementary Figure 1c). These results suggest that the overexpression vector of Gm4419 and Gm4419 knockdown is successfully conducted.

**Figure 2:**
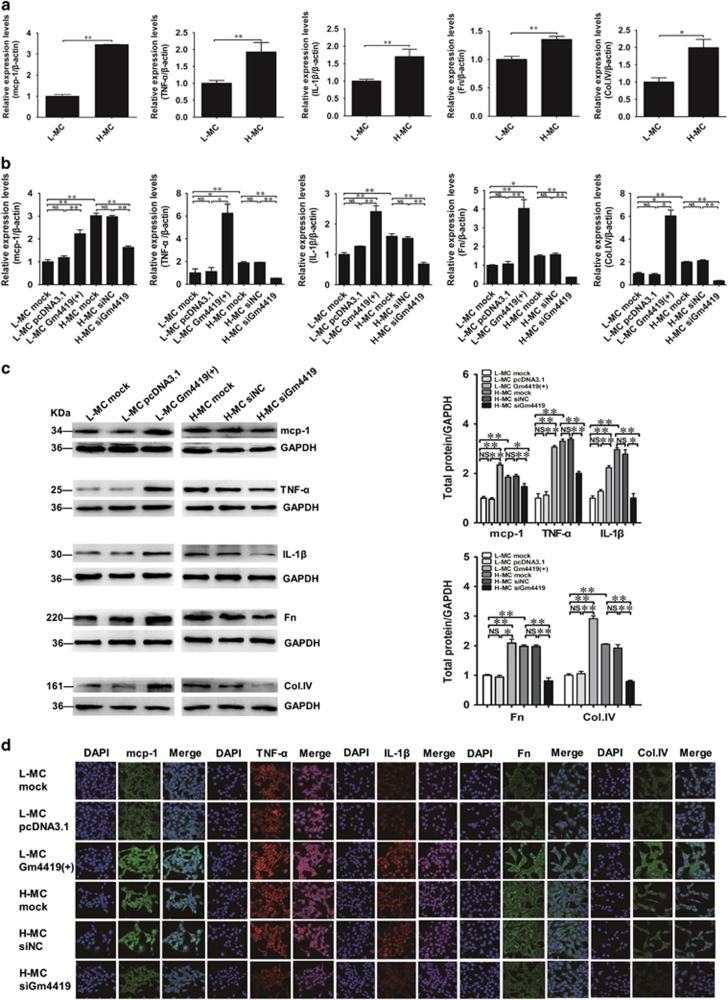

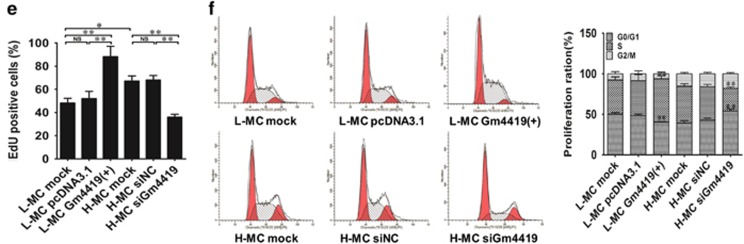
Gm4419 regulates inflammation, fibrosis and proliferation of MCs under high-glucose condition. (**a**) The mRNA levels of pro-inflammatory cytokines (mcp-1, TNF-*α* and IL-1*β*) and fibrosis biomarkers (Fn and Col.IV) in MCs that were stimulated by high or low glucose by qRT-PCR analysis. (**b**) The mRNA levels of pro-inflammatory cytokines and fibrosis biomarkers by qRT-PCR analysis in cells overexpressed or downexpressed Gm4419. (**c**) The protein levels of pro-inflammatory cytokines and fibrosis biomarkers in untransfected or transfected MCs by western blot and quantitative analysis. (**d**) The expressions of pro-inflammatory cytokines and fibrosis biomarkers in untransfected or transfected MCs by immunofluorescent (× 400). (**e**) Proliferative capability of untransfecting or transfecting MCs was analyzed by EdU (5-ethynyl-2′-deoxyuridine) incorporation assay, and the EdU incorporation rate was shown as the ratio of EdU-positive cells to total Hoechst33342-positive cells. (**f**) Untransfected or transfected MCs were analyzed by flow cytometry and quantitative analysis. The percentage of cells in the G0/G1, S and G2/M phases of the cell cycle were calculated. The data are representative of the results of three independent experiments, and the data were presented as means±S.E.M. (*P<0.05, **P<0.01, NS, no significant)

Further, our data showed the expressions of pro-inflammatory cytokines (mcp-1, IL-1*β* and TNF-*α*) and the renal fibrosis biomarkers fibronectin (Fn) and collagen IV (Col.IV) were significantly higher in H-MCs than those in L-MCs by qRT-PCR (Figure 2a). Interestingly, the expressions of the pro-inflammatory and fibrosis biomarkers were increased in L-MCs when Gm4419 was overexpressed (Figures 2b–d). Meanwhile, these pro-inflammatory and fibrosis biomarkers were downregulated in H-MCs when treated with Gm4419 knockdown (Figures 2b–d). Moreover, we observed that the cell proliferation of MCs was obviously suppressed in siGm4419 transfected H-MCs compared with H-MC mock and H-MC siNC by EdU (5-ethynyl-2′-deoxyuridine) incorporation assay and quantitative analysis, but which was reversed with Gm4419 overexpression in L-MC groups (Supplementary Figure 2 and Figure 2e). In addition, flow cytometry results showed that overexpression of Gm4419 increased the percentage of S phase cells and decreased the percentage of G0/G1 phase cells (*P*<0.01, respectively) compared with L-MC mock and L-MC pcDNA3.1, which displayed that cell cycle was accessed into S phase in advance. Although knockdown of Gm4419 in MCs showed the opposite results, which revealed that cell cycle was blocked in S phase, indicating DNA of S phase was damaged (Figure 2f). Therefore, our studies indicate that overexpression of Gm4419 could remarkably promote inflammation, fibrosis and cell proliferation in MCs, and these could be reversed by Gm4419 knockdown.

### Gm4419 regulates the activation of NF-*κ*B signaling pathway by directly interacting with the subunit of NF-*κ*B, p50 in MCs

**Figure 3:**
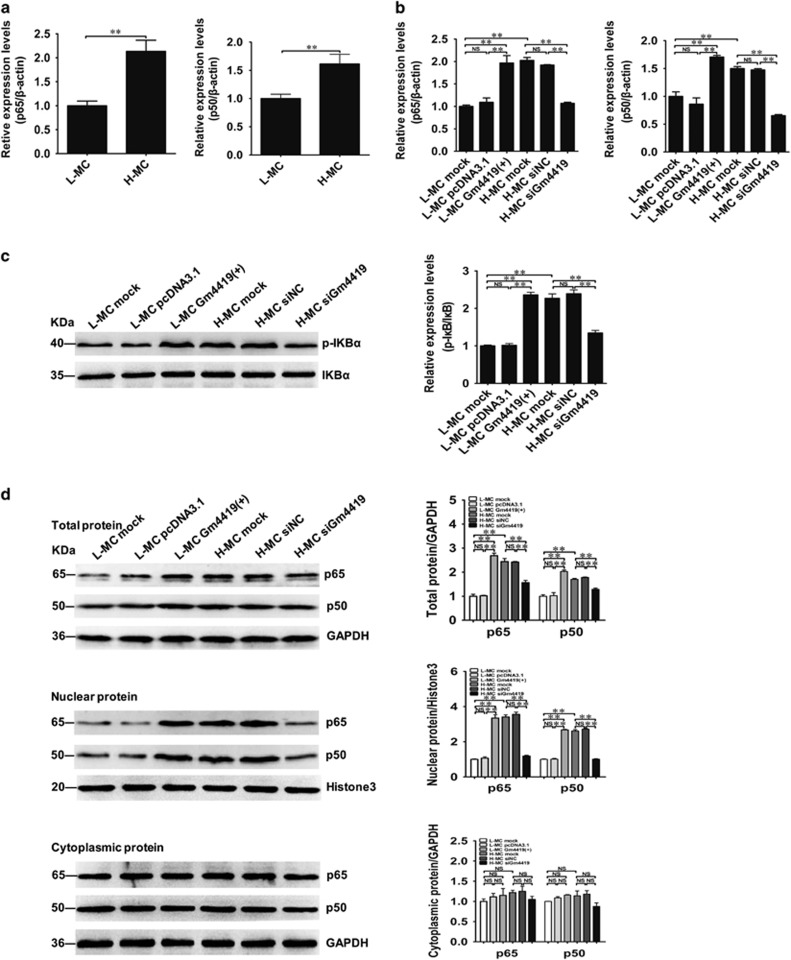

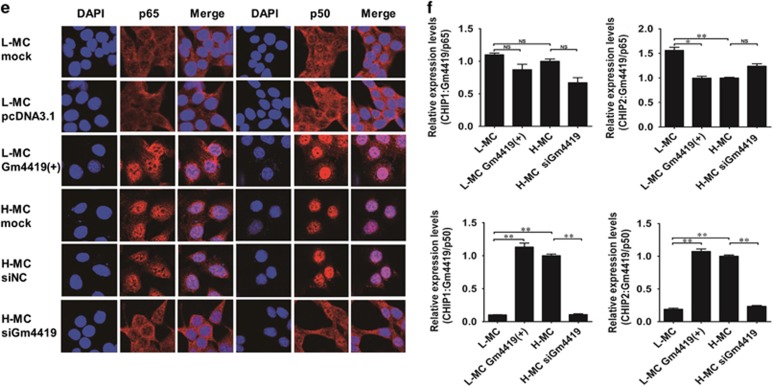
Gm4419 regulates the activation of NF-*κ*B signaling pathway by directly interacting with the subunit of NF-*κ*B, p50 in MCs. (**a**) The mRNA levels of p65 and p50 expression in MCs that were stimulated by were stimulated by high or low glucose by qRT-PCR analysis. (**b**) The mRNA levels of p65 and p50 by qRT-PCR in the L-MC Gm4419 (+) or H-MC siGm4419 group. (**c**) P-I*κ*B*α* and I*κ*B*α* were measured by western blot and quantitative analysis in the L-MC Gm4419 (+) or H-MC siGm4419 group. (**d**) Total protein, cytoplasmic protein and nuclear protein levels of p50 and p65 were measured by western blot and quantitative analysis in cells overexpression or downexpression of Gm4419. (**e**) The regulation of nuclear translocation of p65/p50 by Gm4419 overexpression or knockdown was measured by immunofluorescence staining (× 2800). (**f**) The Gm4419 promoter had a directly transcriptional target for p50 not p65 in mesangial cells by ChIP. The data are representative of the results of three independent experiments, and the data were presented as means±S.E.M. (*P<0.05, **P<0.01, NS, no significant)

To study the role of Gm4419 in NF-*κ*B signaling pathway, the expressions of key molecules in NF-*κ*B signaling including p50, p65, inhibitor I*κ*B*α* and phospho-I*κ*B*α* (p-I*κ*B*α*) were tested in MCs overexpressed or knockdown Gm4419.^25^ Our results showed the expressions of two subunits of NF-*κ*B, p50 and p65 were significantly higher in H-MCs than those in L-MCs by qRT-PCR (Figure 3a). Moreover, overexpression of Gm4419 could increase the expressions of p50 and p65 in L-MCs, whereas Gm4419 knockdown could decrease the expressions of p50 and p65 in H-MCs (Figure 3b). In addition, western blot results showed overexpression of Gm4419 could enhance the expressions of p-I*κ*B*α*, total and nuclear p65/p50, whereas Gm4419 knockdown reduced the expressions of p-I*κ*B*α*, total and nuclear p65/p50 (Figures 3c and d). As known, the I*κ*B*α* phosphorylation and the transfer of p65 and p50 from the cytoplasm to the nucleus were the premise of NF-*κ*B pathway activation. Thus, overexpression of Gm4419 promoted nuclear translocation of p65/p50, whereas Gm4419 knockdown suppressed nuclear translocation of p65/p50 by immunofluorescence (IF; Figure 3e), indicating that Gm4419 could regulate NF-*κ*B signaling activation.

Our above results demonstrated that the change of Gm4419 expression could regulate the expression and activity of NF-*κ*B, then we would further explore the mechanism led to the change of Gm4419 expression. On the basis of the bioinformatics results shown that there were target binding sites with two subunits of NF-*κ*B, p65 and p50 in the promoter region of Gm4419 in Supplementary Table 1, we used ChIP assay to verify the prediction between Gm4419 and NF-*κ*B. Data showed Gm4419 promoter directly interacted with p50, but not p65 (Figure 3f). Therefore, these results indicate that Gm4419 could regulate NF-*κ*B signaling activation via interacting with the subunit of NF-*κ*B, p50.

### P50 synergistically regulates Gm4419 by forming a positive feedback in MCs

**Figure 4:**
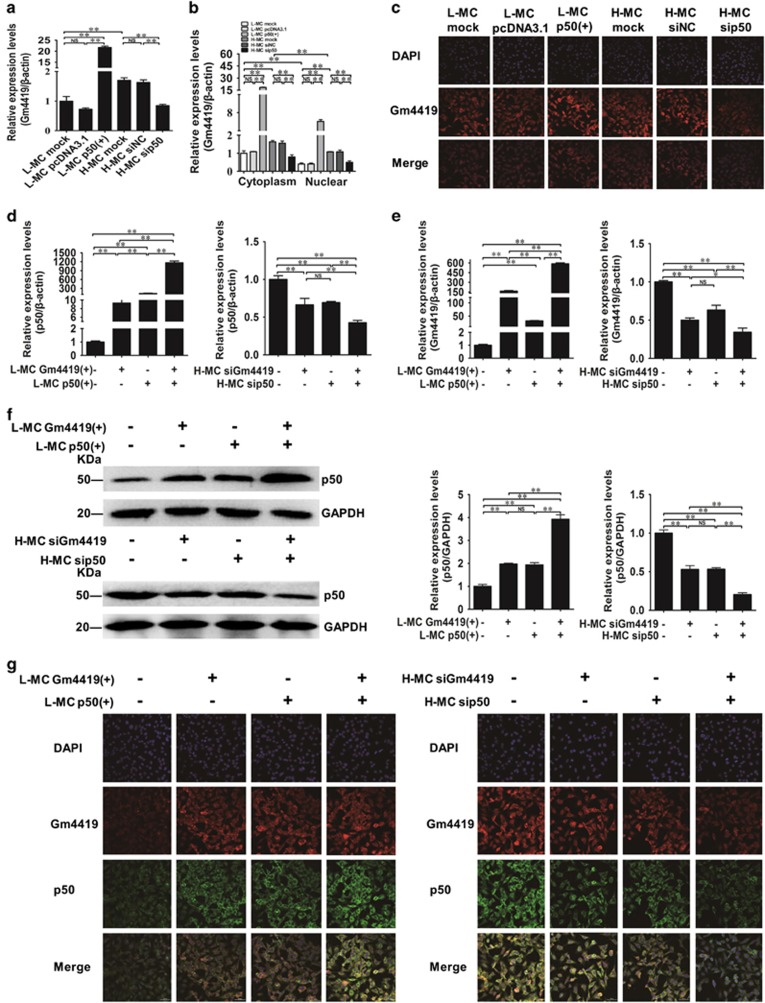
P50 synergistically regulates Gm4419 by forming a positive feedback in MCs. (**a**) Total mRNA levels of Gm4419 expression in cells overexpressed or downexpressed p50 by qRT-PCR. (**b**) The mRNA levels of Gm4419 expression in cytoplasm and nuclear of cells overexpressed or downexpressed p50 by qRT-PCR. (**c**) FISH showed the expression of Gm4419 in cells overexpressed or downexpressed p50 (× 400). (**d**) After co-transfecting with overexpression vectors of Gm4419 and p50 or siRNAs of Gm4419 and p50, the mRNA levels of p50 were detected by qRT-PCR. (**e**) The mRNA levels of Gm4419 tested by qRT-PCR after co-transfection with overexpression vectors of Gm4419 and p50 or siRNAs of Gm4419 and p50. (**f**) P50 in the nucleus was measured by western blot and quantitative analysis in cells co-transfected with overexpression vectors of Gm4419 and p50 or siRNAs of Gm4419 and p50. (**g**) Protein expression of p50 was tested by immunofluorescent staining and RNA expression of Gm4419 was detected by FISH in cells co-transfected with overexpression vectors of Gm4419 and p50 or siRNAs of Gm4419 and p50 (× 400). The data are representative of the results of three independent experiments, and the data are presented as means±S.E.M. (*P<0.05, **P<0.01, NS, no significant)

To further study the effect of p50 on Gm4419, we amplified the full length of p50 using the RACE and cloned the full length of p50 into a pcDNA3.1 vector to construct a stable p50 overexpression plasmid [p50 (+)]. And the plasmid was confirmed by gel electrophoresis experiment and sequence reaction after enzyme digestion (Supplementary Figure 3a). Then, the p50 overexpression plasmid was transfected into L-MCs. Then, qRT-PCR, western blot and IF results showed that p50 (+) largely increased the expression of p50 in mRNA and protein levels when compared with those in L-MC mock and L-MC pcDNA3.1 groups (Supplementary Figures 3b–d). Meanwhile, p50 knockdown was carried out in H-MCs by transfecting with p50 siRNAs as shown in Supplementary Figures 3b–d. As shown in Supplementary Figures 3b and d, the knockdown effect of sip50 (No. 3) was best compared with sip50 (No. 1 and 2) by qRT-PCR, thus we used sip50 (No. 3) for further experiments. By qRT-PCR and FISH, we found that p50 (+) resulted in increased the total, cytoplasmic and nuclear expression of Gm4419 compared with the L-MC mock and L-MC pcDNA3.1 (Figures 4a–c). In addition, p50 knockdown significantly decreased the total, cytoplasmic and nuclear expression of Gm4419 compared with the H-MC mock and H-MC siNC groups (Figures 4a–c). These results indicate that the p50 could regulate Gm4419 expression in MCs. Therefore, combined with the results of Gm4419 regulating the expression of p50, these data suggest that p50 and Gm4419 form a positive feedback to regulate their expressions each other in MCs.

In addition, our results displayed that there was a synergistic effect between Gm4419 and p50. Co-transfecting with of Gm4419 and p50 plasmids into the L-MCs, qRT-PCR, western blot and IF results showed that the expressions of p50 mRNA and protein levels were significantly increased in L-MCs when compared with those in cells transfected with Gm4419 (+) or p50 (+) alone (Figures 4d, f and g). Meanwhile, the expression of Gm4419 levels in L-MCs was significantly increased when compared with that in cells transfected with Gm4419 (+) or p50 (+) alone by qRT-PCR and FISH (Figures 4e and g). In contrast, qRT-PCR, western blot and IF results showed that the expressions of p50 mRNA and protein levels were significantly decreased in H-MCs co-transfected with siGm4419 and sip50 when compared with those in cells transfected with siGm4419 or sip50 alone (Figures 4d, f and g). Also, the expression of Gm4419 level was significantly decreased in H-MCs co-transfected with siGm4419 and sip50 when compared with those in cells transfected with siGm4419 or sip50 alone (Figures 4e and g). There results declare that there is a synergistic effect between Gm4419 and p50 in MCs.

### Gm4419 promotes pro-inflammatory cytokines expression through the NF-*κ*B/NLRP3 inflammasome signaling pathway in MCs

**Figure 5:**
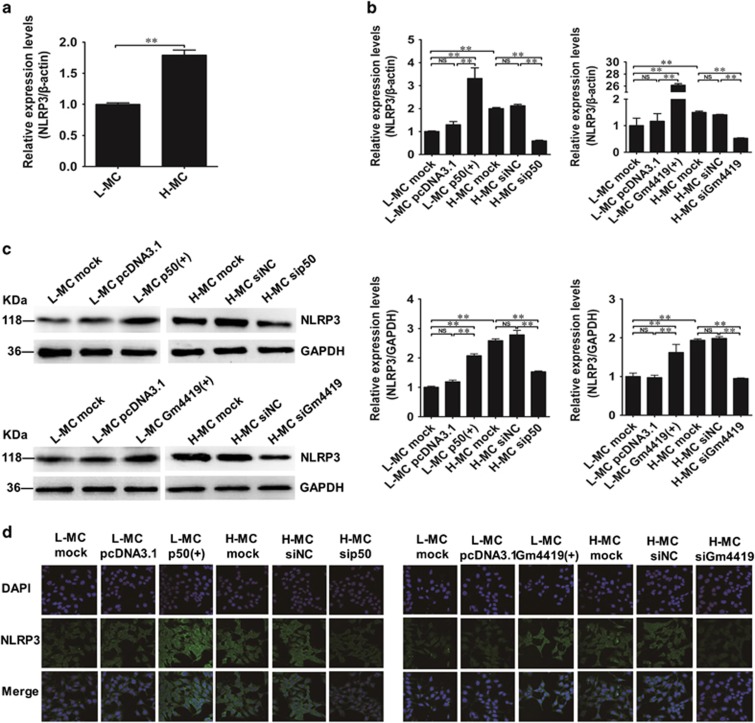
The regulations of Gm4419 and p50 to NLRP3 inflammasome. (**a**)The mRNA levels of NLRP3 inflammasome expression in MCs that were stimulated by high or low glucose by qRT-PCR analysis. (**b**) The mRNA levels of NLRP3 inflammasome were tested by qRT-PCR in cells overexpressed or downexpressed p50 and overexpressed or downexpressed Gm4419. (**c**) Protein levels of NLRP3 inflammasome were tested by western blot and quantitative analysis in overexpressed or downexpressed p50 and overexpressed or downexpressed Gm4419. (**d**) The protein expression of NLRP3 inflammasome by immunofluorescent staining in cells overexpressed or downexpressed p50 and overexpressed or downexpressed Gm4419 (× 400). The data are representative of the results of three independent experiments, and the data are presented as means±S.E.M. (*P<0.05, **P<0.01, NS, no significant)

Recent researches have shown NLRP3 inflammasome have critical role in NF-*κ*B-mediated inflammation in DN.^15, 16^ Our result showed the expression of NLRP3 inflammasome was significantly higher in H-MCs than that in L-MCs by qRT-PCR (Figure 5a). Also, data showed overexpression of p50 could increase the expression of NLRP3 inflammasome in L-MCs, whereas p50 knockdown could decrease the expression of NLRP3 inflammasome in H-MCs (Figures 5b–d). Moreover, we found that overexpression of Gm4419 could increase the expression of NLRP3 inflammasome in L-MCs, whereas Gm4419 knockdown decrease the NLRP3 inflammasome expression in H-MCs by qRT-PCR, western blot and IF (Figures 5b–d). Thus, we hypothesized that NLRP3 inflammasome may participate in Gm4419-induced NF-*κ*B signaling activation in the inflammation of MCs.

**Figure 6:**
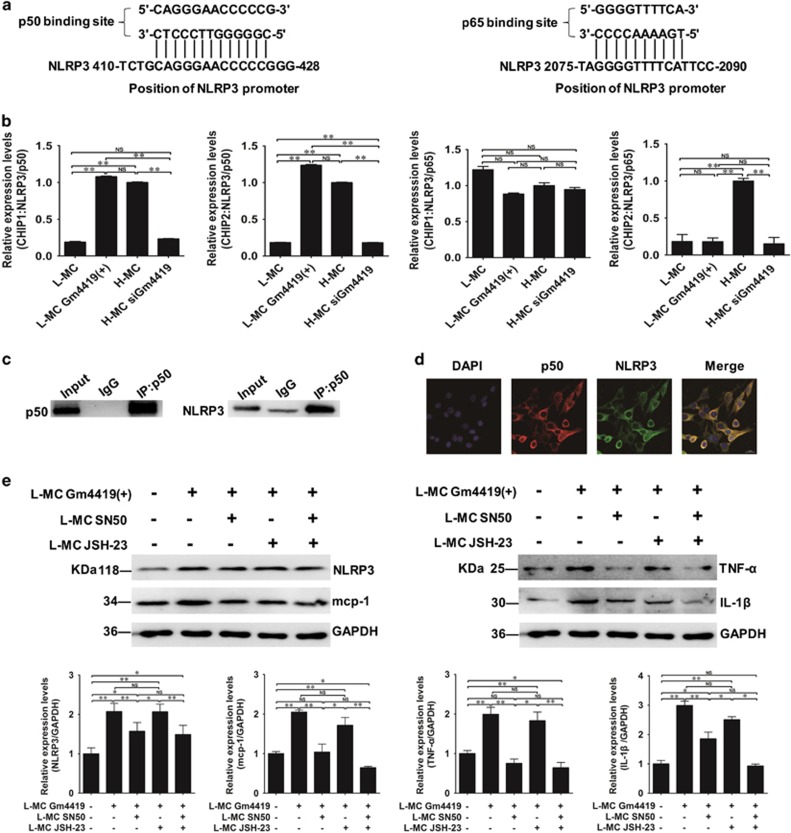
Gm4419 promotes pro-inflammatory cytokines through the NF-κB/NLRP3 inflammasome central inflammatory pathway in MCs. (**a**) The promoter region of NLRP3 inflammasome had potential binding sites to inflammation-related NF-*κ*B (p65/p50) by bioinformatics prediction as presented. (**b**) The NLRP3 inflammasome promoter was a directly transcriptional target of NF-*κ*B/p50 not p65 in mouse mesangial cells with overexpression vectors of Gm4419 or siRNAs of Gm4419 by ChIP. (**c**) The immunoprecipitation was normalized to the amount of p50 pulled down and an IgG light chain (L chain) was used as an input control. (**d**) The co-localizations of endogenic p50 and NLRP3 inflammasome were detected by immunofluorescence. Results showed that endogenic NLRP3 inflammasome and p50 displayed a strong co-localization in MCs (× 800). (**e**) Protein levels of NLRP3 inflammasome and pro-inflammatory cytokines were tested by western blot and quantitative analysis in L-MCs transfected with Gm4419 (+), with or without specific p50 inhibitor SN50 and specific p65 inhibitor JSH-23. The data are representative of the results of three independent experiments, and the data are presented as means±S.E.M. (*P<0.05, **P<0.01, NS, no significant)

To explore the exact relationship between NF-*κ*B and NLRP3 inflammasome in MCs, the interaction was examined by bioinformatics methods and ChIP assay. Our result showed there were NF-*κ*B-binding sites in the NLRP3 inflammasome promoter by online software (Ensembl, JASPAR, TRAP, rVista and TFSEARCH; Figure 6a). Furthermore, ChIP assays demonstrated p50 bound to the NLRP3 inflammasome promoter, but p65 did not (Figure 6b). In addition, to investigate potential interaction between endogenic p50 and NLRP3 inflammasome, immunoprecipitation was performed. Endogenic NLRP3 inflammasome protein was pulled down using anti-p50 antibody. Immunoprecipitates were separated by electrophoresis though SDS-PAGE gels. Results showed that NLRP3 inflammasome protein was successfully immunoprecipitated (Figure 6c). Also, IF results showed that endogenic NLRP3 inflammasome and p50 displayed a strong co-localization in MCs (Figure 6d). Therefore, results demonstrated that p50 have an important role in the NF-*κ*B/NLRP3 inflammasome pathway by directly interacting with NLRP3 inflammasome. To further investigate the role of the two subunits of NF-*κ*B-p50 and p65 in regulating NLRP3 inflammasome pathway in MCs, the p50- or p65-specific inhibitor was used to test the expressions of NLRP3 inflammasome and pro-inflammatory cytokines (mcp-1, TNF-*α* and IL-1*β*). Our data showed overexpression of Gm4419 could not effect on the expression of NLRP3 inflammasome and pro-inflammatory cytokines by co-transfecting with Gm4419 (+)- and the p50-specific inhibitor SN50 in MCs. However, overexpression of Gm4419 still was able to increase the expression of NLRP3 inflammasome and pro-inflammatory cytokines by co-transfecting with Gm4419 (+) and the p65-specific inhibitor JSH-23 in MCs (Figure 6e). Thus, it indicates that it is p50 indispensable for the regulating of Gm4419-mediated upexpression of NLRP3 inflammasome and pro-inflammatory cytokines in MCs. Taken together, these data imply that Gm4419 may regulate the NF-*κ*B/NLRP3 inflammasome inflammatory pathway by p50 that could interact with both Gm4419 and NLRP3 inflammasome in MCs.

## Discussion

To date, studies have reported lncRNAs participated in the DN. Two lncRNAs ENSMUST00000147869 and CYP4B1-PS1-001 have been recently reported to regulate MCs proliferation and fibrosis induced by DN.^26, 27^ Moreover, lncRNAs MIAT and Pvt1 are reported to participate in renal tubular epithelial injury and extracellular matrix accumulation of DN.^28, 29, 30, 31^ Furthermore, single-nucleotide polymorphisms of lncRNA Pvt1 contributes to susceptibility to DN.^32, 33^ However, the exact mechanism of lncRNAs in DN remains mysterious. What is more, the functions of lncRNAs in the inflammatory process of DN have not been reported. In this study, we put forward that Gm4419 directly interacted with p50 to regulate the NF-*κ*B/NLRP3 inflammasome signaling pathway-mediated inflammatory molecular expressions in MCs, and it is associated with the development of inflammatory, fibrosis and proliferation of MCs with high glucose for the first time. Although the discovery of functional lncRNAs represents a new layer of complexity in many human diseases, the functions and mechanisms of lncRNAs during the disease pathophysiological process remain unknown.^34, 35, 36^ In our study, we recently identified that 12 abnormal expression lncRNAs in both renal tissues of db/db DN mice by RNA-seq and qRT-PCR and H-MCs by qRT-PCR, including 9 downregulated and 3 upregulated lncRNAs. In addition, by bioinformatics prediction, we found that only lncRNA-Gm4419 had potential binding sites to DN inflammation-related NF-*κ*B (p65/p50) among the evaluated 12 lncRNAs. Therefore, these suggest that Gm4419 may be an inflammation-related factor in DN.

Gm4419 (Ensembl ID: ENSMUST00000180671) is a lincRNA, which locates in chromosome 12 (Chr12: 21417911-21419803, 1730 bp). However, there is no report about Gm4419 in diseases up to now. As known, lncRNA can perform various functions depending on their subcellular location, if it distributes in the nucleus that mainly involve in the process of transcription and chromatin remodeling, while when it locates in cytoplasmic, which principally participate in gene regulation by forming complexes with specific proteins.^37, 38^ In this study, we found that Gm4419 was a mainly cytoplasmic lncRNA in MCs, indicating that its action in gene regulation. Our studies also found that the expression of Gm4419 was significantly upregulated in MCs cultured under high-glucose condition. In addition, our studies revealed that overexpression of Gm4419 remarkably promoted inflammation, fibrosis and proliferation of MCs with low glucose, whereas silencing of Gm4419 expression led to significant inhibition of cell inflammation, fibrosis and proliferation in MCs with high glucose. Combine with the results of upexpression of Gm4419 in DN, it reveals that the upexpression of Gm4419 represents a potential risk for poor prognosis and contributes to DN development and progression.

Moreover, p50 and p65 form a canonical heterodimer that is known to have a dual role in the activation of NF-*κ*B.^39^ And it is now well known that NF-*κ*B is a key inflammatory pathway in the pathogenesis of DN inflammatory and fibrosis in both animal experiments and clinical studies.^40, 41^ In addition, inhibition of NF-*κ*B activation reduces the expression of inflammatory cytokines to protect the rats against DN.^42^ Also, our previously study have showed that the interaction of NF-*κ*B and miR-451 participates in inflammation of DN.^3^ However, the concrete mechanism still remains unclear. In this study, we found that knockdown or overexpression of Gm4419 could downregulate or upregulate nuclear p65/p50 and effect on the nuclear translocation of p65/p50. It reveals Gm4419 could regulate functional subunits of NF-*κ*B to activate the NF-*κ*B inflammatory pathway. Furthermore, our results further verified that the promoter region of Gm4419 contained a NF-*κ*B/p50 not p65 binding site in mouse MCs by bioinformatics methods and ChIP analysis. In addition, knockdown or overexpression of p50 gene could downregulate or over-regulate the expression of Gm4419 in MCs, suggesting a positive regulatory role for p50 in Gm4419 expression during renal inflammation. As all results above demonstrate that there is an interaction between Gm4419 and p50. Importantly, our work reveals for the first time the involvement of Gm4419 in regulating inflammation by a synergistic effect between Gm4419 and p50 in DN.

Furthermore, previous studies investigated that NLRP3 inflammasome is a novel interesting inflammatory factor in the maturation of IL-1*β* to generate inflammation. And NLRP3 inflammasome has been shown to have a role in the glomerular inflammation, fibrosis and extracellular matrix of DN, which were ameliorated by silencing NLRP3 inflammasome gene.^43, 44^ In addition, recent studies demonstrate that NLRP3 inflammasome participates in NF-*κ*B-mediated inflammatory processes of diseases.^45, 46, 47, 48, 49^ Inhibition of NF-*κ*B suppressed the activation of NLRP3 inflammasome in type 2 diabetes rat.^50^ Moreover, NF-*κ*B is involved in the regulation of NLRP3 inflammasome transcription in TLR-stimulated macrophages through binding to the NF-*κ*B/p65 sequences in the NLRP3 inflammasome promoter.^51^ However, the molecular regulatory mechanisms for relationship between NF-*κ*B and NLRP3 inflammasome are poorly understood in DN. Interestingly, we found that knockdown or overexpression of Gm4419 could downregulated or upregulated NLRP3 inflammasome expression in MCs. Moreover, silencing or overexpression of p60 could reduce or increase the expression of NLRP3 inflammasome. Therefore, these results suggest NLRP3 inflammasome may be involved in the inflammatory effect of Gm4419 mediated by NF-*κ*B. In addition, our results displayed p50 interacted with NLRP3 inflammasome promoter by bioinformatics methods and ChIP analysis. However, p65 did not interact with NLRP3 inflammasome by ChIP analysis. In addition, we found endogenic NLRP3 inflammasome protein was pulled down using anti-p50 antibody. So the co-localization between endogenic NLRP3 inflammasome and p50 displayed that NLRP3 inflammasome and p50 formed a dimer in MCs. Further, our data showed that Gm4419 could not increase the expression of NLRP3 inflammasome in MCs when p50 was blocked by the p50-specific inhibitor SN50. But Gm4419 still worked when p65 was inhibited by the p65-specific inhibitor JSH-23. Therefore, it proves that the subunit of NF-*κ*B, p50, acts as the critical mediator in Gm4419 regulating NF-*κ*B/NLRP3 inflammasome signaling. As known, NF-*κ*B is predominantly found in the form of a heterodimer consisting p65 and p50 subunits,^52^ where p65 exhibits the trans-activation activity via the C-terminal trans-activation domain, whereas p50 directly binds to specific DNA sequence known as NF-*κ*B-binding elements, which co-targets gene promoters in various diseases.^53, 54, 55, 56^ However, earlier study concentrated that LPS-induced p65 binding to the NLRP3 inflammasome promoter in murine macrophages.^51^ The reason for the difference from our result may because of specificity of diseases, tissues and cells. Therefore, p50 may have an important role in the inflammatory of DN. Overall, our data demonstrate that Gm4419 promotes pro-inflammatory cytokines expression through the p50/NLRP3 inflammasome inflammatory signaling pathway in MCs.

In conclusion, this study demonstrates that Gm4419 is a novel NF-*κ*B-associated lncRNA and functions to promote NF-*κ*B (p50)/NLRP3 inflammasome-mediated DN in mouse MCs. Thus, Gm4419 may have a functional role in DN inflammation through NF-*κ*B/NLRP3 inflammasome signaling and may be act as a novel and specific therapeutic target for DN.

## Materials and methods

### Cell culture

Mouse MCs were purchased from the Cell Bank Type Culture Collection of the Chinese Academy of Sciences (Shanghai, China) and they were cultured in complete medium consisting of DMEM containing 2 mmol/l glutamine, 50 mmol/l β-mercaptoethanol, 20% fetal bovine serum (Sangon Biotech, Shanghai, China), penicillin/streptomycin antibiotics and 10 mmol/l HEPES, pH 7.4. Hyperglycaemia is a major stimulus for the development of nephropathy in diabetes patients.^57^ According to the previous results, MCs were stimulated with d-glucose at 5.5 mmol/l glucose plus 19.5 mmol/l mannitol (low-glucose group, L-MC) or at 25 mmol/l glucose (high-glucose group, H-MC) in HERAEUS instrument with 37 °C, 5% CO_2_ and 95% humidity.^3^ HG stimulation had imitated the growth environment of MCs under the condition of DN, and LG stimulation had imitated normal growth environment.

### Small interference RNA treatment

siRNA oligos were used to silence the expressions of Gm4419 and p50. SiRNAs of Gm4419, p50 and scramble were designed and synthesized from Sangon Bio Technology Co Ltd (Shanghai, China), with scramble served as the negative control. The cells were transfected with those oligonucleotides according to the manufacturer's protocol. Briefly, H-MCs were grown in six-well plates and transfected individually with three Gm4419 siRNAs (H-MC siGm4419 no. 1, H-MC siGm4419 no. 2 and H-MC siGm4419 no. 3), three p50 siRNAs (H-MC sip50 no. 1, H-MC sip50 no. 2 and H-MC sip50 no. 3), negative control siRNA (H-MC siNC) and H-MC mock that only maintained transfection reagent without vector at a final concentration of 50 nM per well using Lipofectamine 2000 reagent (Invitrogen, Carlsbad, CA, USA). Because there was no difference between H-MC and H-MC mock groups, mock-transfected cells were used as the control in our study (Supplementary Figure 4). Knockdown effects of Gm4419 and p50 were examined by qRT-PCR using RNA extracted at 24 h after transfection. Cell proliferative capability and the analysis of protein levels at 48 h after transtection were performed using oligos transfected cells. The siRNA sequences will be provided upon request.

### Construction of the overexpression plasmids of Gm4419 and p50 and cell transfection

Using MC's genomic DNA, the identified Gm4419 promoter DNA region was amplified and the PCR products were cloned into the pcDNA3.1 expression vector. The transcription factor gene (p50) overexpression plasmid was constructed as the construction method of Gm4419 plasmids vector. All plasmid transfections used in the experiment were performed at 3000 ng using Lipofectamine 3000 reagent (Invitrogen) according to the established protocol. Briefly, L-MCs were seeded into six-well plates at 5.5 × 10^5^ cells and transfected individually with pcDNA3.1 (+)-Gm4419 [L-MC Gm4419 (+)], pcDNA3.1 (+)-p50 [L-MC p50 (+)], matched empty pcDNA3.1 (+) as negative control [L-MC pcDNA3.1 (+)] and mock that only maintained transfection reagent without vector as control (L-MC mock), Because there was no difference between H-MC and H-MC mock in our study (Supplementary Figure 4). Transfection efficiency was measured by qRT-PCR.

### Bioinformatics analysis of NF-*κ*B-binding sites to the Gm4419 and NLRP3 inflammasome promoters

To study the mechanism of NF-*κ*B and lncRNAs in DN, we predicted the potential transcription factor binding sites between inflammation-related gene NF-*κ*B and the 12 abnormally expressed lncRNAs. Briefly, we preliminary searched the highly conserved region ~2 kb upstream in the promoter regions of lncRNAs and found the promoter sequences of lncRNAs in an online databases (Ensembl: http://asia.ensembl.org/index.html).^58, 59, 60, 61, 62^ Then, the JASPAR (http://jaspar.genereg.net/),^63^ TRAP (http://trap.molgen.mpg.de/cgi-bin/trap_two_seq_form.cgi),^64^ rVista (http://www.rcista.dcode.org)^65^ and TFSEARCH (http://www.cbrc.jp/research/db/TFSEARCH.html)^66^ results revealed that there were NF-*κ*B-binding sites in Gm4419 promoter region. Meanwhile, using the same approach above, the sites of NF-*κ*B binding to the NLRP3 inflammasome promoter were detected.^51^ Our identified potential binding sites were shown in Supplementary Table 2.

### Inhibition of activity of p50 or p65 in cells

The L-MCs were treated with 300 *μ*M-specific p65 inhibitor JSH-23 (Selleck chemicals, Houston, USA) for 24 h^67, 68, 69^ or 50 *μ*g/ml-specific p50 inhibitor SN50 cell permeable inhibitory peptide (Enzo Life Sciences, Farmingdale, NY) for 30–60 min.^70, 71, 72^ However, the detailed time pointof the SN50-treated L-MCs that remained unknown. Our results showed 30 min was the best processing time according to the different time gradients at 30, 60 or 90 min (Supplementary Figure 5).

### Total, cytoplasmic and nuclear RNA extraction and preparation

Total RNA was isolated from MCs using Trizol reagent (Takara, Tokyo, Japan) according to the manufacturer's instructions. The cytoplasmic and nuclear RNA was extracted from MCs using PAKIS Kit (Thermo Fisher Scientific, Waltham, MA, USA) according to the manufacturer's protocol. The purity and concentration of the total RNA were measured using a Nanodrop ND-1000 (Thermo Fisher Scientific) as the manufacturer's operating instructions.

### Real-time reverse transcription (RT)-PCR

An amount of 500 ng of total, cytoplasmic and nuclear RNA was reverse-transcribed into cDNA using Primescript RT Reagent (Takara) in the Gene Amp9700 PCR system (Applied Biosystems, Foster City, CA, USA) under the following conditions: 37 °C for 15 min, 85 °C for 5 s according to the manufacturer's operating instructions. qRT-PCR was used to examine genes levels by Fast Start Universal SYBR-Green Master (Takara) using CFX96 PCR System (Bio-Rad, Foster City, CA, USA). PCR reactions were run in a 10 *μ*l final volume containing 100 ng cDNA, 0.8 *μ*l forward and reverse primers, 5 *μ*l SYBR-Green, and 3.2 *μ*l ddH_2_O. All intermixtures were divided into three parts. The reaction program was initiated at 95 °C for 3 min, followed by 39 cycles at 95 °C for 5 s, 60 °C for 30 s and 72 °C for 30 s, and a final elongation step of 5 s at 65 °C. The primers were designed by primer premier 5.0 and primer BLAST of NCBI in this study and shown in Supplementary Table 2. Relative fold changes of genes expression were calculated by the 2^−ΔΔCT^ method and normalized to *β*-actin.

### FISH

Gm4419 FISH probe was synthesized by Ribo Bio Technology Co Ltd (Guangzhou, China). FISH was performed with the FISH kit according to the manufacturer's protocol (Ribo Bio Tech). Cells were fixed with 4% paraformaldehyde for 10 min at room temperature, and then permeabilized in PBS with 0.5% Triton X-100 on ice for 5 min. Followed by pretreatment with pre-hybridization buffer at 37 °C for 30 min. Subsequently, cells were hybridized with 20 *μ*M using Cy3-labeled RNA of Gm4419 FISH probe mix in a moist chamber at 37 °C overnight. Cells were rinsed thrice in 4 × SSC with 0.1% Tween-20 for 5 min at 42 °C, followed by washing once for 5 min at 42 °C in 2 × SSC and then washed once for 5 min at 42 °C in 1 × SSC. After hybridization, cells were stained with 6-diamidino-2-phenylindole (DAPI) for 10 min at room temperature. Finally, the images were observed with confocal microscope and analyzed with LAS AF Lite (Leica, Solms, Germany).

### ChIP assay

The ChIP analysis was performed using the Millipore EZ-ChIP kit according to the manufacturer's protocol (Millipore, Temecula, CA, USA). In brief, MCs were cross-linked with 1% formaldehyde for 10 min at room temperature after transferred with siGm4419 or Gm4419 (+) and lysed in SDS lysis buffer with 1 mM PMSF. The chromatin was sheared by sonication to an average size of 200–1000 bp. The part of the total lysate was used as ‘Input DNA' control. The chromatin solutions were immunoprecipitated overnight at 4 °C using anti-p50 antibody (Abcam, 1 mg, Carlsbad, CA, USA), or anti-p65 (Abcam, 1 mg), normal rabbit IgG (Millipore, 1 mg) replaced the anti-p50 antibody or anti-p65 antibody as negative control. Salmon sperm DNA-saturated protein G Sepharose was added to protein/DNA complexes for 2 h at 4 °C. The samples that cross-linked by formaldehyde were reversed in 5 mM NaCl at 65 °C overnight after extensive washing. After DNA purification by phenol/chloroform and precipitated with ethanol, the genomic DNA fragments were analyzed by qRT-PCR (primers corresponding to sequences within the promoter regions of Gm4419 and NLRP3 inflammasome were shown in Supplementary Table 3).

### Cell cycle analysis

MCs were seeded into six-well plates at 5.5 × 10^5^ cells. Each group was set three parallel wells. After 48 h transfection, cells were collected by trypsinization, resuspended thrice in cold PBS and fixed with 75% ethylalcohol for 6 h at 4 °C. Finally, the distribution of cell cycle was analyzed by flow cytometry (BD Biosciences, Franklin Lake, NJ, USA).The percentage of cells in the G1-S and S-G2 phases were analyzed using Cell Quest acquisition software (BD Biosciences).

### Cell proliferation assay

The activity of cell DNA replication was analysis by EdU incorporation assay according to the manufacturer's protocol (Ribo Bio Tech). In brief, MCs were cultured in 24-well plates at 1.5 × 10^5^ cells and labeled by EdU buffer for 2 h at 37 °C. Samples were fixed with 4% paraformaldehyde for 30 min at room temperature and then permeabilized in PBS with 0.5%Triton X-100 at 4 °C for 10 min. Followed by staining with Apollo buffer for 30 min at room temperature. Subsequently, cells were stained with Hoechst33342 for 30 min at room temperature. Finally, the images were obtained with microscope (Olympus, Tokyo, Japan) and quantitative analyzed with Image-Pro Plus (Media Cybernetics, Bethesda, MD, USA). The EdU incorporation rate was shown as the ratio of EdU-positive cells (red cells) to total Hoechst33342-positive cells (blue cells).

### Immunofluorescence analysis

Both non-transfected and transfected MCs were fixed with 4% paraformaldehyde for 30 min at room temperature and then permeabilized in PBS containing 0.1% Triton X-100 on ice for 10 min. And then samples were confined by 3% goat serum (Beyotime, Nantong, China) for 1 h at room temperature and incubated by overnight at 4 °C using anti-p50 antibody (Abcam, 1:100), anti-p65 antibody (Abcam, 1:100), anti-NLRP3 inflammasome antibody (Sangon Bio Tech, 1:50), anti-mcp-1 antibody (Sangon Bio Tech, 1:50), anti-TNF-*α* antibody (Bioworld Tech, Minnesote, CA, USA, 1:50), anti-IL-1*β* antibody (Bioworld Tech, 1:50), anti-Fn antibody (Sangon Bio Tech, 1:50) or anti-Col4 antibody (Proteintech, Wuhan, China, 1:50). Then, samples were incubated with dylight488 or dylight549 for fluorescent labeling of goat anti-rabbit antibodies (Thermo Fisher Scientific) for 1 h at 37 °C. Subsequently, cells were stained with DAPI for 10 min at room temperature. Finally, the images were observed with confocal microscope and analyzed with LAS AF Lite (Leica).

### Preparation of proteins (nuclear, cytoplasmic and total protein) and western blot

The MCs were collected after 48 h transfection. Some cells were partitioned into cytoplasmic and nuclear fractions by using nuclear and cytoplasmic protein extraction kit (Beyotime) according to the manufacturer's instructions, and the remaining cells were lysed in PIPA buffer with 1% PMSF (Beyotime) for 30 min on ice, and lysates were centrifuged at 14 000 × *g* for 30 min. Protein quantification analysis was performed using a BCA protein assay kit (Thermo Fisher Scientific). Equal amount of proteins were separated by SDS-PAGE. The integrated densities of the band were quantified by the Image Lab 3.0 software (Bio-Rad) and normalized against a GAPDH internal control. The following primary antibodies that purchased from Abcam and dilutions used were: rabbit polyclonal anti-phospho-I*κ*B*α* (1:600), rabbit polyclonal anti- I*κ*B*α* (1:600), rabbit polyclonal anti-p65 (1:600) and rabbit polyclonal anti-p50 (1:600). The primary antibodies that obtained from Bioworld and dilutions used were as follows: rabbit polyclonal anti-TNF-*α* (1:500) and rabbit polyclonal anti-IL-1*β* (1:500). The primary antibodies were purchased from Sangon Bio Technology Co Ltd and diluted: Rabbit polyclonal anti-NLRP3 inflammasome (1:500), rabbit polyclonal anti-mcp-1 (1:500) and rabbit polyclonal anti-Fn (1:500). Rabbit polyclonal anti-Col4 (1:500) was obtained from Proteintech Co Ltd.

### Immuoprecipitation analysis

MCs were seeded into six-well plates at 5.5 × 10^5^ cells and cultured with HG DMEM medium for 24 h. Cells were collected and lysed in PIPA buffer with 1 mM PMSF (Beyotime) for 30 min on ice, and lysates were centrifuged at 14 000 × *g* for 30 min. Subsequently, the p50/NLRP3 inflammasome complexes were immunoprecipitated from 200 *μ*g of protein by using anti-p50 antibody (2 *μ*g), anti-IgG (2 *μ*g) antibody and protein A plus G agarose beads (20 *μ*g), followed by western blot for the protein levels of p50 and NLRP3 inflammasome. The immunoprecipitates were washed for four times with PIPA buffer. Then, the pellet was resuspended in loading buffer and incubated at 100 °C for 5 min before SDS-PAGE to release the proteins from the beads.

### Statistical analysis

Statistical analyses of the data were performed by SPSS software (Version 20.0, SPSS, Chicago, IL, USA). The difference between two groups was performed with *t*-test for functional analysis, and statistical significance was determined with one factor analysis of variance in no less than three groups. A probability value of *P*<0.05 was regarded as the criterion of statistical significance and presented as mean±S.E.M. comparisons. Statistical analyses were performed using the GraphPad Prism software (GraphPad Software, San Diego, CA, USA).

## Figures and Tables

**Figure 7:**
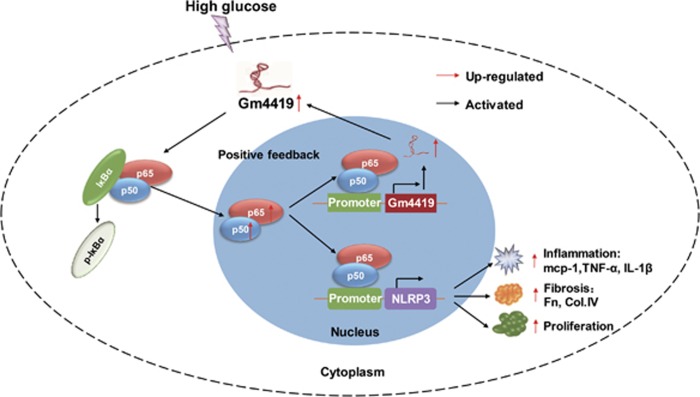
A schematic representation of the proposed model: possible mechanism of Gm4419 via the NF-*κ*B/NLRP3 inflammasome central inflammatory pathway induced inflammation, fibrosis and proliferation under high-glucose conditions. Gm4419, which increased in high-glucose levels, upregulated nuclear NF-*κ*B (p65/p50) expression and nuclear translocation from the cytoplasm to the nucleus, led to an enhanced nuclear NF-*κ*B expression and NLRP3 inflammasome activation, and induced p50 binding to Gm44419 and NLRP3 inflammasome promoter regions. Subsequently, nuclear p50 promoted the transcription of Gm4419, increased the expression of nuclear Gm4419 that raised the expression of Gm4419 in cytoplasm of cells. Thus, they formed a positive feedback in Gm4419/NF-*κ*B signaling. In the meantime, p50 promoted transcription of NLRP3 inflammasome and downstream genes: pro-inflammatory cytokines including mcp-1, TNF-*α* and IL-1*β*, and fibrosis biomarkers including Fn and Col.IV

**Table 1:** Twelve DN-related lncRNAs with the same expressional tendencies in RNA-seq as well as qRT-PCR of renal tissues and qRT-PCR of MCs

*Expressional tendencies*	*lncRNAs*	*RNA-Seq and RT-PCR in renal tissues of DN mice*	*RT-PCR in MCs with high glucose*	
Upregulation	Gm4419	2.86	1.63	2.83
	6030443J06Rik	1.89	1.86	1.44
	1500026H17Rik	1.81	1.787	1.71
Downregulation	Snhg18	1.66	1.85	1.51
	2610035D17Rik	5.87	4.63	5.91
	C330002G04Rik	4.25	14.22	2.01
	C920006O11Rik	19.55	38	2.08
	4930556M19Rik	1.74	2.01	5.10
	D630029K05Rik	2.18	2.56	4.90
	Snhg6	4.47	7.53	3.47
	1700020I14Rik	1.44	1.28	2.72
	AV099323	2.72	1.83	1.67
