# Supplementary material for: LincRNA-Gm4419 knockdown ameliorates NF-κB/NLRP3 inflammasome-mediated inflammation in diabetic nephropathy
Source: Cell Death Dis. 2017 Feb 2;8(2):e2583–. doi: 10.1038/cddis.2016.451 (PMC5386454; doi:10.1038/cddis.2016.451)
Supplement: Supplementary Information [file cddis2016451x1.docx]

**Supplement Figure 1** The construction and verification of over-expression Gm4419 plasmid vector and Gm4419 siRNAs. (**a**) Gm4419 over-expression plasmid was authenticated by gel electrophoresis and sequencing after restriction enzyme digestion. (**b**) QRT-PCR detected that cells in L-MC group which transfected with the Gm4419-pcDNA3.1 (+) vector [Gm4419 (+)] showed a large increment in expression of Gm4419 when compared with those transfected with mock or empty pcDNA3.1 (+) vector [pcDNA3.1 (+)]. Also, qRT-PCR data showed that cells in H-MC group which transfected with the siRNAs (no.1, no.2 and no.3) when compared with those in H-MC mock and H-MC siNC groups. Moreover, siRNA Gm4419 (no.3) show a largest reduction in expression of Gm4419. (**c**) FISH data showed the expression of Gm4419 was over-expressed in L-MC Gm4419 (+) group while it was down-expressed in H-MC siGm4419 group. The results were consistent with the results of qRT-PCR (x 200). The data are representative of the results of three independent experiments, and the data are presented as means ± SEM (*p < 0.05, **p <0.01, ^NS^ no significant).

**Supplement Figure 2** Proliferative capability of MCs over-expressed or down-expressed Gm4419 was analyzed by EdU incorporation assay (x200). Red cells: EdU positive cells, blue cells: Hoechst33342 positive cells.

**Supplement Figure 3** The construction and verification of over-expression p50 plasmid vector and p50 siRNAs. (**a**) NF-κB/p50 over-expression plasmid was authenticated by gel electrophoresis experiment and sequence reaction after restriction enzyme digestion. (**b**) QRT-PCR detected that [p50 (+)] could significantly over-regulate the expression of p50 in L-MC group, while siRNA of p50 (no.3) could down-regulate the expression of p50 in H-MC group. (**c**) P50 in the nucleus and cytoplasm were measured by western blot and quantitative analysis in L-MC p50 (+) or H-MC sip50 (no.1, no.2 and no.3) groups. (**d**) Nuclear translocation of p50 with L-MC p50 (+) or H-MC sip50 was measured by immunofluorescence staining (x 800). The data are representative of the results of three independent experiments, and the data are presented as means ± SEM (*p < 0.05, **p <0.01, ^NS^ no significant).

**Supplement Figure 4** (**a**) The mRNA levels of Gm4419 expression in L-MC, L-MC mock, H-MC and H-MC mock by qRT-PCR. (**b**) The mRNA expression of p50 in L-MC, L-MC mock, H-MC and H-MC mock by qRT-PCR. The data are representative of the results of three independent experiments, and the data are presented as means ± SEM (*p < 0.05, **p <0.01, ^NS^ no significant).

**Supplement Figure 5** The time gradients of L-MCs in SN50 were measured by western blot and quantitative analysis. The data are representative of the results of three independent experiments, and the data are presented as means ± SEM (**p <0.01, ^NS^ no significant).
