# Supplementary material for: LincRNA-Gm4419 knockdown ameliorates NF-κB/NLRP3 inflammasome-mediated inflammation in diabetic nephropathy
Source: Cell Death Dis. 2017 Feb 2;8(2):e2583–. doi: 10.1038/cddis.2016.451 (PMC5386454; doi:10.1038/cddis.2016.451)
Supplement: Supplementary Information [file cddis2016451x2.docx]

**Supplement Figure 1**


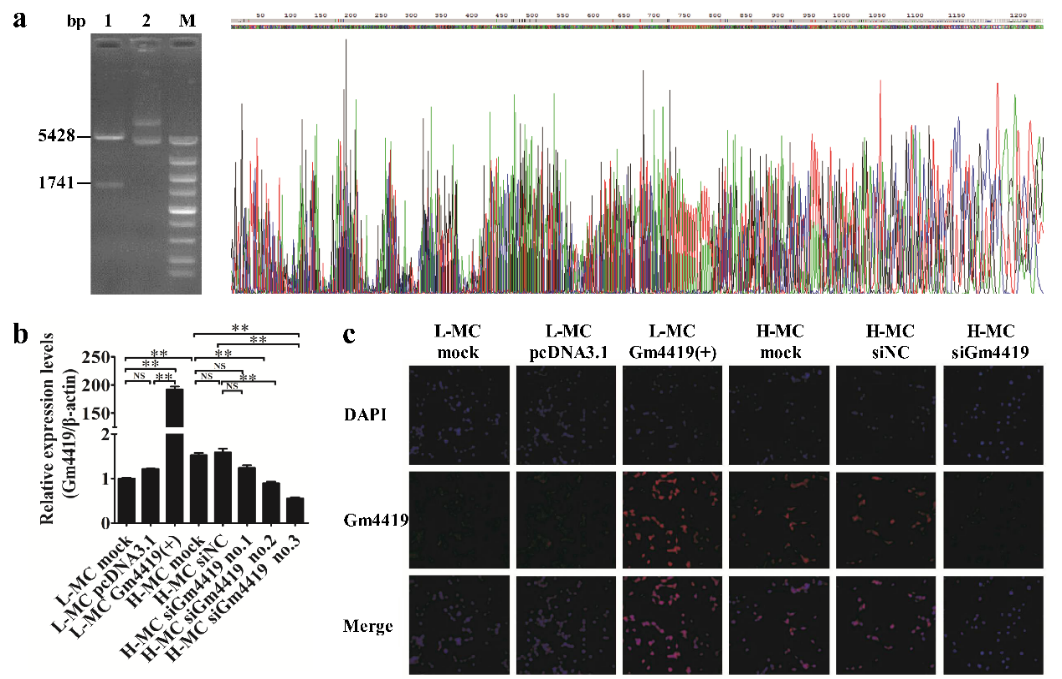


**Supplement Figure 2**


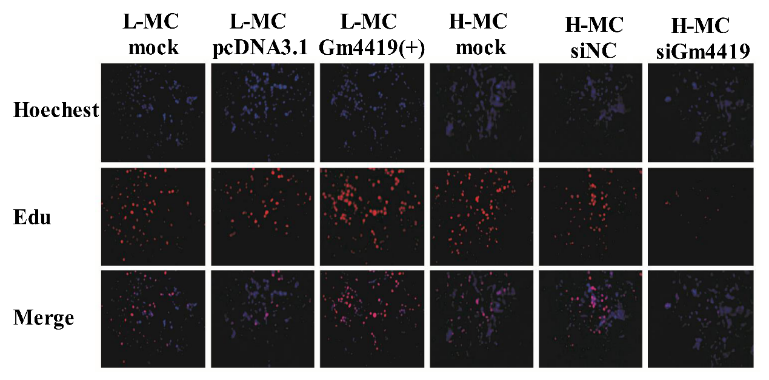


**Supplement Figure 3**


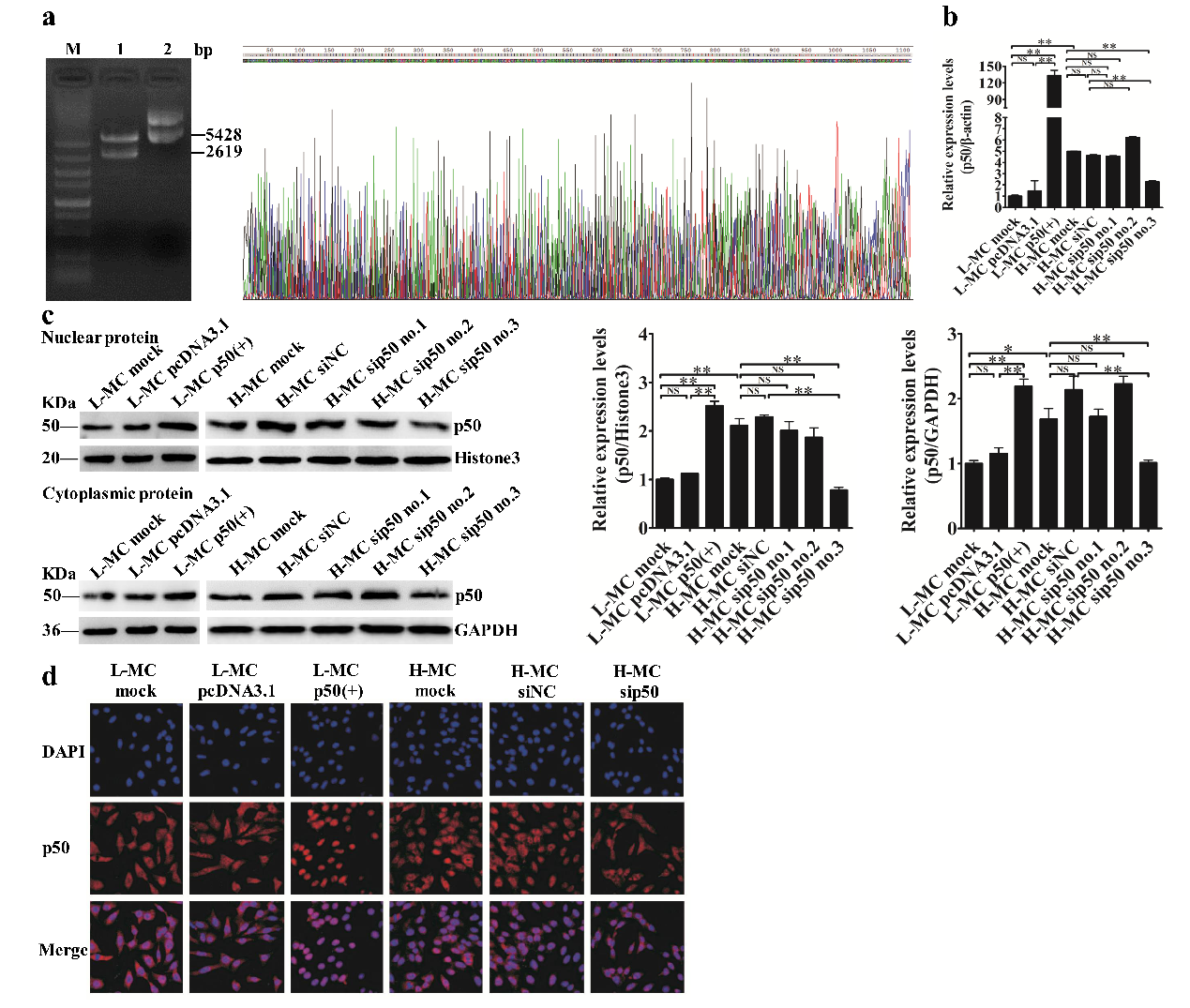


**Supplement Figure 4**

**
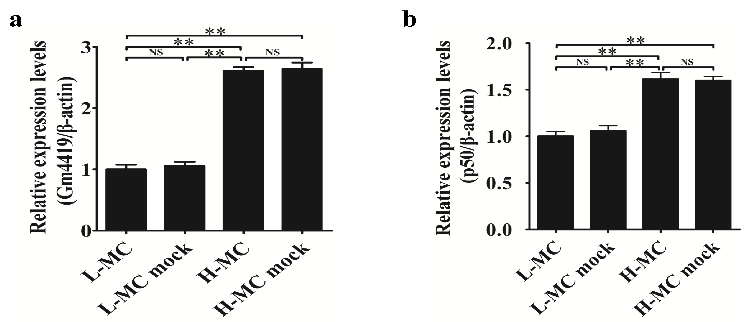
**

**Supplement Figure 5**

**
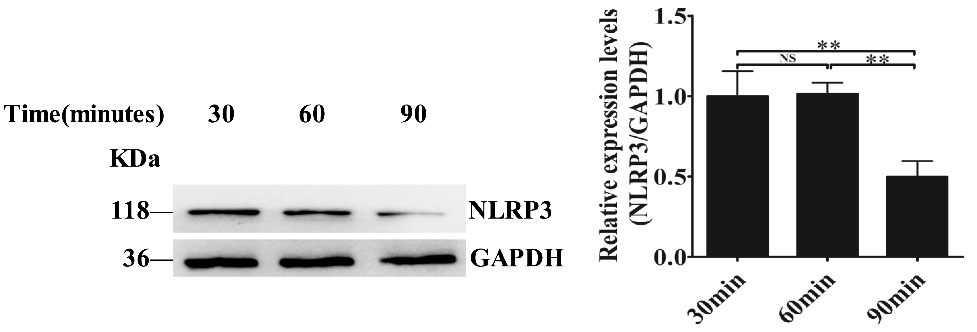
**
