# Supplementary material for: LincRNA-Gm4419 knockdown ameliorates NF-κB/NLRP3 inflammasome-mediated inflammation in diabetic nephropathy
Source: Cell Death Dis. 2017 Feb 2;8(2):e2583–. doi: 10.1038/cddis.2016.451 (PMC5386454; doi:10.1038/cddis.2016.451)
Supplement: Supplementary Information [file cddis2016451x3.docx]

**Supplementary Table 1** Bioinformatics prediction of the potential binding sites between NF-κB and 12 lncRNAs.

| lncRNAs | Location | Length(bp) | NF-κB/p65 binding sites to lncRNAs | NF-κB/p50 binding sites to lncRNAs | |
| --- | --- | --- | --- | --- | --- |
| Gm4419 | Chr:12:21417910-21419803 | 1730 | 478-CGGGGTTTCC-487 | | 478-CGGGGTTTCCT-488 |
| Snhg18 | Chr:15:32240568-32244662 | 1482 | No | | No |
| C920006O11Rik | Chr:9:78175913-78178879 | 1444 | No | | No |
| C330002G04Rik | Chr:19:23037389-23075853 | 2054 | No | | No |
| 4930556M19Rik | Chr:15:10714835-10790123 | 4029 | No | | No |
| D630029K05Rik | Chr:10:116956823-116972609 | 2133 | No | | No |
| Snhg6 | Chr:1:9908637-9944118 | 473 | No | | No |
| 2610035D17Rik | Chr:11:113043894-113201838 | 1436 | No | | No |
| 1700020I14Rik | Chr:2:119594296-119607502 | 8300 | No | | No |
| AV099323 | Chr:2:132253356-132261333 | 921 | No | | No |
| 6030443J06Rik | Chr:5:22550436-22807850 | 4299 | No | | No |
| 1500026H17Rik | Chr:10:89686370-89700866 | 3027 | No | | No |

Chr: Chromatin

**Supplementary Table 2** Primers for qRT-PCR detection of 14 lncRNAs, NF-κB, NLRP3 inflammasome, inflammatory cytokines and fibrosis biomarkers.

| Genes | Primers(5’ – 3’ ) | Genes | Primers(5’ – 3’ ) |
| --- | --- | --- | --- |
| Gm4419 | Forward: GGAACCAAGCAGACCGAAGAC  Reverse: CCCCAACCCACAGGAACATAA | 1700020I14Rik | Forward: TCCTGGTTCTTCCATCCTGT  Reverse: ACGGCTTTCCTGTGTTGAGT |
| 4930533I22Rik | Forward: GCTGCCTCAGTCTCTTTGGA  Reverse: GCTCAAGTGTTTGTCCTCATCA | 6030443J06Rik | Forward: TGATGGAAAGTCAGTGGGATT  Reverse: TCAAAGGATGGAAATGTGTCTG |
| Snhg18 | Forward: AAATGCCCTCTTCCTGAGTG  Reverse: GTGCTCCTTCTTGTGGGTGT | 1500026H17Rik | Forward: GACCTCTCCAGACACACTCTCCTG  Reverse: AATCATTTTCCTTACTTCCCACCA |
| D630029K05Rik | Forward: TTGCCGTGCTTCTCTTTCTT  Reverse: CTGGGTGCTTGTTTGAGGTT | AV099323 | Forward: TGAGCAGGAAACAAACATACAACC  Reverse: ACTTTAATGCCTCGCCTTCAATAG |
| Snhg6 | Forward: AGGTGGCTGTAGTGGATGTC  Reverse: TGGCTTCTTGACTTTGTTCA | 4930556M19Rik | Forward: CTGGGCGAATGTTTGTTTCT  Reverse: GCCTTCTGTGGAACTCACCT |
| 2610035D17Rik | Forward: CTTCTTCTGTTTCGGGCTGT  Reverse: GTTTGTAGTCGGGCAAGGAT | RP23-341H6.1 | Forward: GCACTGTTGGCTCCTGTTGT  Reverse: CATCACTCCGCACGACTTC |
| C920006O11Rik | Forward: GGGCATCTCCCTTACCTGTT  Reverse: CCTCTCGTTTCCCATTTCTG | C330002G04Rik | Forward: CATCAATACCCTCCGTCCAA  Reverse: GCCTTCTCAACTTCCTGGTG |
| p65 | Forward: GACCTGGAGCAAGCCATTAG  Reverse: CACTGTCACCTGGAAGCAGA | p50 | Forward: CAGGTCCACTGTCTGCCTCT  Reverse: GGAAGGATGTCTCCACACCA |
| NLRP3 | Forward: AGTGGATGGGTTTGCTGGGAT  Reverse: TGCGTGTAGCGACTGTTGAGG | mcp-1 | Forward: GACCCGTAAATCTGAAGCTAATGC  Reverse: AATTAAGGCATCACAGTCCGAGTC |
| TNF-α | Forward: CCCTCCTGGCCAACGGCATG  Reverse: TCGGGGCAGCCTTGTCCCTT | IL-1β | Forward: CATCAGCACCTCACAAGCAGA  Reverse: TGGGGAAGGCATTAGAAACAG |
| Fn | Forward: TCTGGGAAATGGAAAAGGGGAATGG  Reverse: CACTGAAGCAGGTTTCCTCGGTTGT | Col.IV | Forward: TGGTCTTACTGGGAACTTTGCTGC  Reverse: ACCCTGTGGTCCAACGACTCCTCTC |
| β-actin | Forward: GGCTGTATTCCCCTCCATCG  Reverse: CCAGTTGGTAACAATGCCATGT |  |  |

**Supplementary Table 3** List of primers used for ChIP-qPCR determination of DNA-binding of NF-κB (p65/p50)

| Genes | binding sites | Primers (5’ – 3’ ) |
| --- | --- | --- |
| Gm4419/p65 | -487~ -478 | Forward (no.1): GCTCGGGTAGGTTCGGTTGG  Reverse (no.1): TGGTTGCTGCTACAGGACACG  Forward (no.2): TCGGGTAGGTTCGGTTGGC  Reverse (no.2): CGGGGAGGATGAGGGGAGT |
| Gm4419/p50 | -488~ -478 | Forward (no.1): GCTCGGGTAGGTTCGGTTGG  Reverse (no.1): TGGTTGCTGCTACAGGACACG  Forward (no.2): TCGGGTAGGTTCGGTTGGC  Reverse (no.2): CGGGGAGGATGAGGGGAGT |
| NLRP3/p65 | -2086~ -2077 | Forward(no.1): GTGTTTCTGATAACTTCGTTTTCTGG  Reverse(no.1): TGCAACGGACACTCGTCATCTT  Forward(no.2): GTGTTTCTGATAACTTCGTTTTCTGG  Reverse(no.2): TCTCGGGGCTTAGGTCCACA |
| NLRP3/p50 | -426~ -414 | Forward(no.1): GCTGAGCCCTGAGGTTTCACTT  Reverse(no.1): TGTCAAAGACGGATTGGCAGTT  Forward(no.2): CTTGCCACTGCTTATGTCCCC  Reverse(no.2): TTTTCCAAACCCAAGCAATAGAG |
